# Educational Leave as a Time Resource for Participation in Adult Learning and Education (ALE)

**DOI:** 10.3389/fpsyg.2019.02977

**Published:** 2020-01-22

**Authors:** Fabian Rüter, Andreas Martin, Josef Schrader

**Affiliations:** German Institute for Adult Education (DIE), Bonn, Germany

**Keywords:** educational leave, time resources, rational choice, policy implementation research, evaluation

## Abstract

The study investigates effects of the implementation of a law authorizing educational leave in Germany on individual participation in adult learning and education (ALE). In 2015, the federal state of Baden-Württemberg introduced the so-called *Bildungszeitgesetz*, legitimating an exemption for eligible employees of up to 5 days per year with continued payment of salary. Explaining participation in ALE is a central subject of educational research at national and international level. Current theoretical assumptions of rational choice and empirical findings of educational and socio-statistical research suggest that within the general population, individuals’ availability of time affects the decision to participate and therefore lastly determines participation in ALE. However, current academia mainly discusses time as either a prerequisite for learning activities or as an observable outcome of participation and not as an explanatory factor. Furthermore, since recent studies remain on a descriptive level regarding influences of time on participation in ALE, no causal effects of the availability of time on participation are estimated. Hence, our study addresses this research gap by investigating effects of educational policy interventions such as the *Bildungszeitgesetz* on participation in ALE. Policy interventions are ideally suited to examine the significance of time resources for participation, as the implementation of the *Bildungszeitgesetz* provides a specific timeframe for employees to participate in ALE outside of their working time. Drawing on data from the German National Educational Panel Study, we employ a difference-in-differences estimation strategy with propensity score matching and instrumental variable to identify the direct causal effect of the implementation of the *Bildungszeitgesetz* on participation in ALE (*N* = 709). This combination toward causal inference controls for observed and unobserved baseline differences as well as heterogeneous treatment effects. The results reveal a non-significant but heterogeneous treatment effect of the implementation of the *Bildungszeitgesetz* on individual participation in ALE. Contrary to our theoretical assumptions derived from rational choice approaches, we cannot confirm the hypothesis that the availability of time resources due to the implementation of the *Bildungszeitgesetz* causes a positive effect on participation in ALE. Furthermore, the results reveal that the implementation causes decreasing participation rates for younger adults, women and significantly for migrants.

## Introduction

Explaining participation in adult learning and education (ALE) is a central subject of educational research both at the national level in Germany (Leber and Möller, 2007; Kuper et al., 2017) and from an international comparative perspective (Rubenson et al., 2006; Egetenmeyer, 2016). Current research on determinants of participation in ALE points to a complex interaction between structural context conditions and individual characteristics (Kaufmann and Widany, 2013). According to Boeren et al. (2010), participation in an educational activity requires a successful match between the demand for adult education on the level of individuals and companies and the institutionalized offer provided by educational institutions. Current educational and socio-statistical research primarily focuses on explaining societal inequalities und selectivity in participating in ALE with socio-demographic, -economic and -cultural factors (Behringer, 1999; Boudard and Rubenson, 2003; Hubert and Wolf, 2007; Kuper et al., 2017). Although socio-statistical characteristics can explain inequalities and participation selectivity to some extent, factors correlating with these characteristics and the decision to participate in an educational activity remain unexplained. This is because not only do social-statistical characteristics determine individual participation in ALE, but rather the varying expected utilities attributed to these factors and available resources (Walter and Müller, 2014).

In adulthood, “time rivalries emerge between work, family and recreation on one hand and learning on the other” (Schmidt-Lauff and Bergamini, 2017, p. 157). Because learning and education are inextricably linked to time (Faulstich, 2006; Schmidt-Lauff, 2018), as time is a prerequisite for any kind of learning activity, these time constraints are particularly relevant during adulthood, since no established *time institutions* (Geißler, 1999; Garhammer, 2001) like compulsory schooling exist.

Generally speaking, time for education can arise from parts of individuals’ work or recreation time, but also from a combination of both times (Dobischat and Seifert, 2003). In the relationship between working time and personal time (Nowotny, 1995), time becomes a scarce resource for participation in ALE. From the individual perspective, different life situations and varying positions in the employment system describe specific time rivalries that affect educational participation due to the varying availability of time resources. Thus, structural time rivalries depend not only on the position in the employment system, but also on age and gender; biographical time rivalries depend on different phases of life such as career entry, career advancement, starting a family or retirement (Schmidt-Lauff, 2008, 2018). Therefore, time rivalries result from multi-layered backgrounds and relations (Schmidt-Lauff and Bergamini, 2017). Hence, we can define time as a scarce resource with biographical and structural variation, determining individual participation in educational activities due to its availability. Time as a scarce resource can therefore be understood as a factor of social inequality with regard to individual participation ALE (Jurczyk, 1998; Alhadeff-Jones, 2010).

Because of time as a scarce resource, we assume heterogeneous influences of the availability of time resources on individual participation in ALE. Despite the interdependence of time and participation in ALE, we observe a paradox in the political and academic discussion on the relevance of time. Although both politics and academia emphasize the importance of time for participation in ALE and call for lifelong learning as a holistic time regime (OECD, 2001; Boeren et al., 2010; CEDEFOP, 2012), time as an explanatory factor of individual participation has so far been neglected and underestimated in empirical research. With regard to the subject of time, we can distinguish three central perspectives in current research.

In the first perspective, time spent in ALE is the observable result of individual or company-related decision-making processes on educational participation and selection processes in an educational activity. This perspective is based on surveys such as the Adult Education Survey (AES) and the Continuing Vocational Training Survey (CVTS). Kaufmann and Widany (2013) assume that participation in ALE is realized through self-selective and external-selective processes in different opportunity structures. These processes and thus the selection in ALE are determined by various factors at the micro, meso and macro level (Boeren et al., 2010). This perspective is limited to measuring outcomes via hours of time spent in ALE, while the underlying decision-making processes remain unexplained.

In addition, the AES provides variables focusing on time constraints where on the one hand the variable time is not explicitly used, but which on the other hand can be used as a reliable proxy. On a descriptive level, the results of the AES 2016 in Germany reveal that despite their time-intensive employment as well as biographical and structural time rivalries (Schmidt-Lauff, 2008, 2018) regarding the access to educational activities, employees are the most active group of adults participating in ALE. This observation relates to the fact that 71% of every educational activity in ALE is fully or at least partially paid by the employer in Germany in 2016 (Schönfeld and Behringer, 2017). Furthermore, empirical findings of the AES highlight that employees working full-time show higher participation rates in ALE (49%) compared to part-time employees (40%) (Bilger and Strauß, 2017). Current research explains this difference in participation rates with varying investments of monetary and time resources in ALE by the employer. A common finding is that employers expect comparatively higher returns from investments in ALE for full-time employees than from investments in part-time employees (Hubert and Wolf, 2007; Kuper and Kaufmann, 2010).

The second perspective on time as a barrier for ALE participation identifies subjectively rated time rivalries that result in limited opportunities to participate, which in turn ultimately lead to non-participation. In addition to costs and expected utilities of participation in ALE, individual reasons to participate in ALE or not become relevant in the decision-making process (Bilger and Käpplinger, 2017). Research on barriers shows, that ‘not having enough time’ becomes an escape route in the justification of non-participation (OECD, 2011; CEDEFOP, 2012; Schmidt-Lauff, 2018). Furthermore Radovan (2012) makes the criticism that it is not possible to generalize the results from different studies on barriers because of different methodologies used and non-comparable theoretical approaches. Moreover, since educational reports such as the AES or CVTS remain on a descriptive level, no causal effects of subjectively evaluated barriers on participation can be estimated.

The third perspective focuses on working time and analyzing the effects of time on participation based on time spent at work. In this perspective, it is not a question of training time, but of working time. Therefore, we do not present empirical findings for this perspective at this point.

At this point, we can conclude that influences on individual participation in ALE due to available time resources remain widely unexplained in current research, as scholars mainly discuss time either as a prerequisite for learning activities or as an observable outcome of participation in ALE. This research gap on effects of time in general and of available time resources on participation in particular challenges empirical research to develop new research strategies, theoretical assumptions and methodological approaches. The scarcity and, at the same time, the great significance of time resources discloses the urgency of analyzing presumably heterogeneous influences of time resources on participation in ALE beyond participation rates, time volumes and barriers.

One possibility of analyzing these influences and estimating the corresponding effects is to treat time as an explanatory variable. This includes questions of how individual participation in ALE changes due to the availability of time resources and how actors in the adult educational system can govern or regulate the availability of time. Schrader (2011) describes the system of continuing education as a multi-level system consisting of the level of educational governance (macro-level), the level of organization institutions (meso-level) and the level of teaching and learning processes (micro-level). Public policy can regulate or influence the interaction between offer (meso-level) and demand (micro-level) “by reducing the indirect costs, by offering services, and by other means” (Boeren et al., 2010, p. 47).

German educational policy primarily discusses the topic of time with regard to laws on educational leave. The concept of paid educational leave is part of the political debate on establishing an adults’ right to participate in education (Luttringer and Pasquier, 1980; Bahnmüller, 2002; Schmidt-Lauff, 2005). While most policies on the labor market focus on structures and financing of ALE such as offering vouchers (Witte, 2009; Käpplinger et al., 2013) or the implementation of quality management systems (Hartz and Meisel, 2011; Schrader and Jahnke, 2014), laws on educational leave put a specific focus on time. These laws ought to regulate the legal entitlement of employees to participate in ALE. Eligible employees have the legal option to apply to accredited courses or educational institutions in vocational, general or political education or for qualification as a volunteer.

One way of investigating effects of time on participation in ALE is the use of working time as learning time, legitimized by a legislative option in form of laws on education leave. In 2015, the German federal state of Baden-Württemberg introduced such a law called the *Bildungszeitgesetz*, legitimating an exemption for employees of up to 5 days per year with continued payment of salary. The implementation of this law makes it possible to investigate the effectiveness of the implementation: It allows us to estimate causal effects of such educational policy interventions on individual participation in ALE while simultaneously investigating effects of time on participation in ALE.

## Theoretical Background

### Theoretical Explanation of Individual Participation in ALE

With regard to the importance of time as a resource for individual action and the underlying decision-making processes, current research shows a major lack of knowledge when explaining individual participation in ALE due to time constraints and the availability of time resources. In order to investigate such influences, our theoretical considerations need to explain possible effects of the availability of time on the individual decision to participate in an educational activity. Thus, our theoretical approach focuses on self-selective processes rather than external selective processes by the employer. We introduced time as a scarce resource varying in biographical and structural dependence at the individual level (Schmidt-Lauff, 2008, 2018) and as an element of indirect costs (Bellmann and Leber, 2017) to be raised in order to participate in an educational activity. Hence, we assume significant but heterogeneous influences of available time resources on the decision to participate in ALE.

In order to make these assumed influences empirically accessible, we build our theoretical assumptions on action theories (Esser, 1993, 1999; Kroneberg, 2005, 2011). Economic approaches like human capital theory (e.g., Becker, 1964) explain participation in ALE with self-selective processes as a result of a rational-economic investment (Kaufmann and Widany, 2013). In the context of economic, but also social and educational sciences, the rational choice (RC) approach has a long tradition and covers a variety of theoretical models and theories (Blossfeld and Müller, 1996; Blossfeld and Prein, 1998). Although the theoretical approaches framed by the collective term RC vary, we can identify some general assumptions underlying all approaches. According to Diekmann and Voss (2004), the nomological core assumption of every RC theory is that individuals choose the best alternative possible on the basis of situational conditions, valuations and expectations, in which they can achieve the highest expected utility. This implies, that individuals always try to achieve maximum welfare while at the same time having minimum costs when pursuing preferences or goals (Allingham, 2002). The individual decision to act in a certain way, “therefore, can be seen as being based on a cost-benefit analysis” (Boeren et al., 2010, p. 48).

Although RC is widely established in current research, it still faces criticism regarding the assumption of rationality and maximization of utility (Yee, 1997; Boudon, 2003; Hodgson, 2003; Eriksson, 2011). In every RC theory, the definition of the situation (Esser, 1999) as well as social norms, values and emotions do not represent independent explanatory factors of individual behavior (Elster, 1989; Yee, 1997; Kroneberg, 2005). Instead, RC attempts to reconstruct any action as rational in terms of a cost-benefit analysis (Münch, 1998; Opp, 1999).

Based on this criticism, newer theoretical approaches by Kroneberg (2005, 2011) intend to identify exact conditions and action-generating mechanisms that lead to rational decisions and at the same consider that individual behavior can be guided and determined by relevant social norms, values, communication-oriented discourses or emotions. Furthermore, the objective of these approaches is to take into account that individuals act differently in seemingly identical situations. With reference to more recent experimental studies based on community games and the prisoners’ dilemma (Larrick and Blount, 1997; Kay and Ross, 2003; Kay et al., 2004; Liberman et al., 2004; Stocké, 2004) the author refers to the situational volatility of goals, preferences and expectations in a given situation. Referring to these studies, individual goals and preferences within a situation can no longer be treated as a constant factor. Consequently, the central idea of Kroneberg (2005, 2011) is that in some situations, individuals make a deliberate and thus rationally calculating choice of action whereas in other situations, they follow certain routines, norms or emotions unquestioningly. Kroneberg (2005, 2011, 2014) refers to this phenomenon as the ‘variable rationality’ of individuals.

An integrative theory of action in which the “important sociological insight that the definition of the situation matters” (Lindenberg, 1989, p. 194) and in which the variable rationality are systematically taken into account in the explanation of individual behavior is the model of frame selection (MFS) by Kroneberg (2005, 2011). Kroneberg bases his theoretical assumptions on the frame selection theory (FST) by Esser (1996, 2001). Central elements of the MFS are frames and scripts, which Kroneberg (2011) refers to as mental models based on approaches of cognitive social psychology and cultural anthropology (DiMaggio, 1997). While frames are mental models of situations, scripts represent mental models of sequences of actions (Moskowitz, 2005; Kroneberg, 2014). The MFS intends to explain how individuals interpret and define a situation they are facing (frame selection), which program of action they activate (script selection) and which action they are willing to perform (action-selection).

Central to the MFS is furthermore the idea of a mode selection, which distinguishes the processes of frame-, script- and action-selection either in an automatic-spontaneous mode (as-mode) or a reflecting-calculating mode (rc-mode) (Kroneberg, 2005, 2011). This differentiation bases on the idea of a *variable rationality* that is taken from dual-process theories in social psychology (Fazio, 1990; Chaiken and Trope, 1999). The as-mode represents a selection of action that is solely based on a strongly activated script, meaning that individuals do not weight any costs and benefits of different alternatives. Scripts can represent moral norms, conventions, routines, and emotional or cultural reaction schemes. In contrast to the as-mode stands the rational-calculating mode. The rc-mode represents a selection based on a deliberate choice, in which an individual systematically processes consequences and their probabilities. An individual who thinks about which action matches a defined situation best generally does so in the context of practical interests (Kroneberg, 2011). Furthermore, individuals choose the alternative that maximizes the subjectively expected utility out of a feasible set of alternative actions in the rc-mode. Consequently, the rc-mode is modeled by using the subjective expectancy-value theory or SEU-theory (Esser, 1999).

This differentiation of modes raises the question under which conditions individuals act automatic-spontaneous or rational-calculating. The additional value of the MFS is the specification of these conditions, “thereby endogenizing an actor’s degree of rationality” (Kroneberg, 2014, p. 98). Kroneberg identifies four variables that determine the mode of information processing: opportunities, motivation, effort and accessibility. The mode selection formalizes the relationship between these four determinants. An “automatic-spontaneous mode becomes more likely, the fewer the *opportunities* and the lower the *motivation* for conscious deliberation, the greater the *effort* necessary for this mental activity, and the higher the *accessibility* of a ready-to-use program.” (Kroneberg et al., 2010, pp. 8–9). In contrast to this, the rc-mode becomes more likely, the greater the motivation to reflect is, and the more individual abilities and situational efforts allow reflection (opportunities). Motivation is thus an important factor, as the rc-mode is associated with a higher effort of time and energy. When there is no high motivation to reflect, individuals will select frames, scripts and action more spontaneously (Kroneberg, 2011). “Conversely, human beings seem to engage in a more effortful and more comprehensive mode only if it seems necessary, possible, and profitable to do so” (Kroneberg et al., 2010, p. 9).

Kroneberg (2005, 2011) generally assumes that the action-selection is structured and affected by the definition of the situation. Frames and scripts activate specific knowledge, goals, values and emotions. Taking the theoretical assumption of influences of frames and scripts on the action-selection into account, the modeling in terms of the SEU-theory looks as follows. The set of action alternatives *A*_*k*_, the expectations *p*_*m*_ as well as the valuations *U*_*m*_ are each represented as a function of the selected frame *F*_*i*_ and script *S*_*j*_(Kroneberg, 2011):

$$S\,E\,U\,\left(A_{k}|F_{i},S_{j}\right)=\sum p_{m}\,\left(F_{i}\,S_{j}\right)\,U_{m}\,\left(F_{i}\,S_{j}\right)\,f\,o\,r\,e\,v\,e\,r\,y\,A_{k}\in \left(F_{i}\,S_{j}\right)$$

As a result, the MFS allows expecting and systematically describing influences of the frame- and script-selection on the actions-selection. Nevertheless, Kroneberg (2011) states, that prior to further theoretical specifications, the MFS does not contain any empirically testable hypotheses about how these influences of the selected frames and scripts on the actions-selection look like.

Having introduced the core elements of MFS, it needs to be answered, which assumptions we can conclude with regard to the decision to participate in ALE and possible effects of time on this specific decision. Regarding the decision to participate in ALE, we can refer to the frame- and script-selection as the “building of a behavioral intention” (Kroneberg, 2014, p. 99). The action-selection is then the performance or realization of this intention to participate in an educational activity and is the observable result of this decision-making process. Regarding the action-selection, we cannot assume that the decision to participate in an educational activity is the result of an automatic-spontaneous process. Participation in ALE is rather a deliberate decision and consequently a break with everyday routines. This becomes obvious in using time resources from the ratio of working and personal time. Since we cannot assume a spontaneous action when participating in ALE, we build our theoretical assumptions regarding the effect of time on the decision to participate on the rc-mode.

The action-selection in the rc-mode characterizes, that individuals compare different alternatives, resulting consequences and the subjective probability of occurrence of these consequences. A decision is therefore based on a reflection process taking different consequences of different alternatives into account. The MFS formalizes this process by applying a decision rule taken from the rational choice approach. For each course of a feasible set of alternatives, individuals evaluate possible outcomes and combine them with the subjective expectation to realize the expected utility. Thereby, possible outcomes are combined with the assumed effectiveness of each alternative course of action to weight each course. By defining an exact decision rule based on the constructs of expectancy and value, the theory allows a causal explanation of individual action (Esser, 1999). The calculation of the SEU-weight of each alternative course of action bases on the function *SEU*(*A*_*i*_) = ∑*p*_*ij*_^∗^*U*_*j*_. Esser (1993) assumes, that “every actor weights each alternative of action *A*_*i*_ concerning every goal *U*_*j*_ with the associated subjective propability *p*_*ij*_“ (Esser, 1993, p. 10). The result of this evaluation is the “total subjective expected utility of alternative*A*_*i*_” (Esser, 1993, p. 10) of a feasible set of alternatives. According to this calculation, individuals choose the alternative with the highest SEU-weight (Esser, 1993, 1999).

Kroneberg (2011) states, that the SEU-theory as a RC approach can be applied to specify the action-selection in the rc-mode. Central to the MFS is that the interpretation of the action-selection modeled by the SEU-theory differs from the initial SEU-theory. According to the initial SEU-theory by Savage (1954), subjective expectations of the utility of an alternative are formed rationally. In contrast to this, the MFS interprets the SEU calculation psychologically as a modeling of a reflected decision-making process. By applying the SEU-theory, we can explain the action-selection to participate in an educational activity in the rc-mode completely as a result of a cost-benefit analysis (Kroneberg, 2011).

Using the MFS, we can identify two concrete aspects in which time as a mechanism influences the decision to participate in ALE. First, the reflection in the rc-mode costs time and energy. Furthermore, time provides opportunities to reflect the given situation. Second, time is an element of indirect costs of participation in ALE, which are represented in the cost-benefit function of the SEU-theory. Based on this theoretical model, we can derive empirically testable hypotheses that we test by applying a difference-in-difference estimation strategy.

### Laws on Educational Leave – National and International Perspectives

The idea of paid educational leave goes back to the Convention of the International Labour Conference (ILO) in 1974. According to Schmidt-Lauff and Bergamini (2017), as of 2017, 35 countries have ratified the ILO convention. In an international comparative perspective, laws on educational leave are regulated at various levels in a multilevel governance of training (Heyes and Rainbird, 2011), including legislations at the national or regional level, collective or transnational arrangements (CEDEFOP, 2012). The main objective of any instrument on educational leave is to provide a specific timeframe for employees that enables them to participate in an educational activity outside of their working time (Schmidt-Lauff and Bergamini, 2017). Laws on educational leave provide a legal entitlement to learning time for eligible employees, while at the same time imposing obligations on the employer regarding the exemption and continuing payment of salary (Grotlüschen and Haberzeth, 2018). In addition, laws on educational leave enable and empower eligible employees to select themselves in educational activities. Participation thus becomes independent of the external selective logic by the employer and different opportunity structures arise through self-selective processes in educational activities.

With regard to Germany and the introduction of paid educational leave, an implementation of a general federal law has not yet taken place. As of now, the German government does not currently see any need for introducing a uniform legislation in Germany (Deutscher Bundestag, 2011). Nevertheless, though there is no common legislation on educational leave in Germany, currently 14 out of 16 federal states have introduced own laws on educational leave. These laws ought to regulate the legal entitlement of employees to participate in ALE, primarily in vocational (CVET), political and general education or for qualification in volunteering and legitimate a paid exemption of approximately 5 days per year (Grotlüschen and Haberzeth, 2018). With regard to the content of ALE, there has been a clear trend in recent decades away from political education toward CVET (BMBF, 2006; Reichling, 2014). The financing of participation in ALE due to the entitlement of educational leave is provided by a mixed funding model (Friebel, 1993a,b) in which the employee pays the participation costs and the employer pays the continued payment of salary during the period of exemption. In the majority of federal states, the participation rate is according to Reichling (2014) approximately less than one percent. Higher participation rates of up to three percent are approximated in Lower Saxony, Rhineland-Palatinate and Bremen. These low participation rates reveal that educational leave appears to have a socially selective effect on participation in ALE (Wagner, 1996; BMBF, 2006; Jäger, 2007).

Research on the topic of educational leave has been realized since the late 1970s both on the national level in Germany (Kejcz et al., 1979; Hindrichs et al., 1984; Bremer, 1999) and at the international level (Luttringer and Pasquier, 1980; Bryant, 1983; Schütze, 1983; Morrissey and Mcnamara, 2005; Gould, 2016; Oh et al., 2016). Generally speaking, there are hardly any empirical studies or at least systematic analyses or observations in Germany (Grotlüschen and Haberzeth, 2018). An exception are the most recently published results on the revision of the law on educational leave in the German federal state of Bremen in 2010 by Robak et al. (2015b). A central statement of the authors is that there is no current research on educational leave in Germany and as a result, there is only limited knowledge on developments of participation rates due to laws on educational leave with regard to the development of participation-rates in ALE. In an integrative research design, the authors investigate influences of the revision on various levels and actors. They focus on developments of the organization program, the planning of this program as well as on the adults participating in ALE who are empowered by the law on educational leave in Bremen. Furthermore, they interviewed works councils about the realization of educational leave in their companies. The study is an *ex post* analysis study using qualitative and quantitative survey methods. In this study, no causal treatment effects are estimated and the results remain on a descriptive level.

The overview of the current and partly almost historical state of research regarding laws on educational leave shows considerable research gaps in understanding the motivation to participate, didactics and methods applied in courses in educational leave and especially the effectiveness of these laws (Schmidt-Lauff, 2005; Siebert, 2015). Generally speaking, participation in educational leave is approximately relatively low and insufficiently documented in Germany. Some federal states provide no statistical documentation while other federal states only provide incomplete data or have completely discontinued statistical documentation (Grotlüschen and Haberzeth, 2018). Available statistical data from different federal states differ in the way the data were documented and collected. On the one hand, the number of applied courses are monitored at the administrative level of the federal state; on the other hand, educational institutions provide statistical information on the number of participants applicable to the law on educational leave. In addition, information on participation in educational leave differs according to age and gender. Moreover, in some statistics, the number of participants is even undifferentiated regarding individual characteristics (Deutscher Bundestag, 2011). As a result of the incomplete documentation on participation in educational leave, neither any general statements on the effectiveness of laws on educational leave in Germany can be derived, nor can the current situation as a whole be presented systematically (Grotlüschen and Haberzeth, 2018).

Laws on educational leave intend to foster and support self-selective processes in segments of ALE, which we can describe with regard to the AES as job-related or non-job-related non-formal education and training. Referring to the descriptions in the AES, we can identify individual characteristics that influence the likelihood of participation in the segments of ALE that laws on education leave intend to foster and support. Furthermore, we can contrast these conditions and effects of individual characteristics to participate in employer-sponsored non-formal education and training and therefore participation that is realized through external-selective processes. While this description does not allow us to identify target groups or participants in educational leave, it allows us to identify individual characteristics that influence participation in the segments of ALE that laws on educational leave intend to foster and support.

In this perspective, the AES distinguishes participation in the segments of employer-sponsored job-related non-formal education and training (external-selective processes) as well as in job-related and non-job-related non-formal education and training (self-selective processes). Results of the multivariate analyses of the AES (Kuper et al., 2017) reveal significant differences regarding the conditions and individual characteristics like gender, migration background, formal vocational qualification and age to participate in either segment of education and training. Thereby, the AES provides causal explanations of participation in either segments. First, the findings reveal a statistically significant negative effect of male gender on the likelihood to participate in job-related as well as in non-job-related non-formal education and training. In contrast to this, the results reveal that there is no significant gender difference in the likelihood of participation in employer-sponsored job-related non-formal education and training. This result remains non-significant even after controlling for context characteristics regarding the company and employment contract. Second, the results highlight a statistically significant positive effect of the migration status of immigrants on participation in job-related and non-job-related non-formal education and training. Referring to the AES, these results are explained with the fact, that because women as well as adults with a migration background and immigrants experience an increasing number of discontinuous employment biographies, they participate to a higher level in job-related non-formal education and training to obtain knowledge or to learn new skills needed for a current or future job. The statistically significant higher probability of participation in non-job-related non-formal education and training of immigrants compared to Germans without a migration background may refer to specific needs (languages) or obligations (integration courses) of this population (Kuper et al., 2017). In comparison to this, the results regarding participation in employer-sponsored job-related non-formal education and training reveal a statistically significant lower likelihood of participation for employees with a migration background and immigrants. Third, further conditions for the likelihood of participation in either segment of education and training are the formal vocational qualification and the required vocational qualifications for the type of occupation. On the one hand, the formal vocational qualifications influence the likelihood of participation in job-related non-formal education and training; on the other hand, the required vocational qualifications for the type of occupation significantly effect the likelihood to participate in employer-sponsored job-related non-formal education and training. As the level of required vocational qualification increases, so does the likelihood of participation. Lastly, with regard to the effect of age on the likelihood to participate in either segment, the result reveal that adults aged 35 to 44 years show the highest participation rate in job-related as well as in employer-sponsored education and training. In contrast to this, the results reveal the lowest participation rate for this age group in non-job-related non-formal education and training.

### Implementation of the *Bildungszeitgesetz* in Baden-Württemberg in 2015

In 2015, the *Bildungszeitgesetz* was introduced in the German federal state of Baden-Württemberg legitimating an exemption of up to 5 days per year with continued payment of salary. The objective of the state government is to support and foster the willingness of employees in Baden-Württemberg to participate in ALE. In order to achieve this goal of higher participation rates in ALE, the implementation of the law on educational leave is considered as an effective intervention (Landtag von Baden-Württemberg, 2015). According to the legislation, eligible employees are those whose main working place is located in Baden-Württemberg and whose employment status exists for at least 12 months at the same company (§4 BzG BW). The law ought to regulate the legal entitlement of employees to participate in vocational (CVET) and political education as well as for qualification in volunteering. At the level of educational institutions, educational programs may only be offered in accredited organization institutions. The accreditation by the regional council requires, that the institution exists for at least two years, that courses are systematically planned, organized and offered and t that the institution certifies the quality of the educational work by a quality certificate (§9 BzG BW). The implementation of the *Bildungszeitgesetz* also includes an evaluation mandate to investigate the effectiveness of the law (§ 9 BzG BW).

According to the official evaluation of the law (f-bb, 2019), approximately 4,765,600 employees are eligible according to §2 BzG BW. The aim of the study was to evaluate whether the objectives of the implementation of the *Bildungszeitgesetz* are actually achieved. To answer this general question, the authors employed qualitative and quantitative survey methods to survey the actors involved: Participants (*N* = 484), eligible employees (*N* = 535), companies (*N* = 498), educational institutions (*N* = 724), and stakeholders (*N* = 10). The results of the evaluation assume that approximated 1.12% of eligible employees in Baden-Württemberg apply to the *Bildungszeitgesetz* every year. The data basis for calculating the percentage of persons who actually applied to the *Bildungszeitgesetz* is the number of eligible employees in Baden-Württemberg. Although the study above takes a first step at investigating the effects of implementing laws on educational leave, the effectiveness of the implementation cannot be systematically evaluated and assessed based on the conducted surveys.

First, there are no official statistics and documentation of both the application to the *Bildungszeitgesetz* with regard to participants and educational institutions, as there is no form of obligatory reporting in Baden-Württemberg. Due to selective and non-representative data, the evaluation does not provide any statistically reliable evidence on the effectiveness of the implementation of the *Bildungszeitgesetz* on participation in ALE. In fact, no official statistics and representative survey data exist that can provide reliable information regarding the real number of eligible employees who have made use of the *Bildungszeitgesetz* in Baden-Württemberg. Second, the number of approximately 4,765,600 eligible employees is not adjusted for the number of employees whose employment status does not yet exist for 12 months. Thus, the statistical population includes employees who are eligible as well as those who are not. The estimation of the participation rate therefore bases on unadjusted data. Third, the evaluation does not perform an estimation of changes of individual participation in ALE caused by the implementation of the law, as the statistical analyses are not conducted in a counterfactual design. Therefore, no causal treatment effects of the implementation on individual participation are estimated. A central result of the authors themselves is that it is difficult to conclude within the framework of the *ex post* analyses whether there is an actual increase in participation in ALE as a result of the implementation of the *Bildungszeitgesetz* (f-bb, 2019).

In comparison to other existing laws on education leave in German federal states, the *Bildungszeitgesetz* in Baden-Württemberg differs with regard to the definition of eligible employees, content of courses and the duration of the exemption in days. As a result, we cannot transfer any assumptions regarding the approximated effectiveness of the implementation of the *Bildungszeitgesetz* from the current state of research and laws on educational leave in other federal states.

## Research Questions and Hypotheses

Regarding the significance of time on the decision to participate in ALE, current research calls for more research on effects of time on educational activities (Sellin and Elson-Rogers, 2003; Schmidt-Lauff and Bergamini, 2017).

We use the implementation of the *Bildungszeitgesetz* in Baden-Württemberg (2015) as an educational policy intervention, thus as a treatment, to investigate the question of influences of time on individual participation in ALE. According to the action-selection in the rc-mode formulated in the MFS (Kroneberg, 2005, 2011), we can conclude the following implications regarding influences of time on the decision to participate in ALE by applying the SEU-theory. In order to participate in an educational activity, individuals need to invest time resources out of the ratio of working and personal time. Thereby, time represents an element of resulting indirect costs (Bellmann and Leber, 2017) of an educational activity for which according to Esser (1999) there is a negative interest. Therefore, time affects the subjectively expected cost-benefit analysis regarding participation in ALE and the subjective probability to achieve the expected benefits. When more time is available for participation in an educational activity at the individual level, then the increased level of control of time has a positive effect on the expected cost-benefit analysis of participating in ALE. As the subjectively expected cost-benefit analysis increases, so does the likelihood of a decision to participate.

We operationalize individual participation in ALE as the number of attended courses in ALE, whereby we only include courses that fit to the contents covered by the *Bildungszeitgesetz*. An operationalization of participation in ALE as the volume of hours spent in educational activities is not possible because of incomplete information in the NEPS data set. Consequently, we cannot test treatment effects on the time (in hours) spent in ALE. In consequence, our hypothesis regarding the effect of the implementation of the *Bildungszeitgesetz* on individual participation in ALE is:

Hypothesis 1: The implementation of the *Bildungszeitgesetz* has a positive effect on the number of attended courses in ALE among the eligible employees in comparison to those adults, who are untreated by the law.

In addition to this general hypothesis, we can a second hypothesis on the effect of the implementation of the *Bildungszeitgesetz* from the SEU-theory. In the subjective cost-benefit analysis, time as an element of indirect costs would have to be particularly weighted and evaluated as valuable by individuals that objectively have scarce time resources. The subpopulations of interest are identified by their marital status and by the existence of children in their household. Due to the implementation of the *Bildungszeitgesetz*, these subpopulations should benefit in particular from the legal option to use working time to participate in ALE. Our hypothesis regarding effects of the implementation of the law for these subpopulations is therefore:

Hypothesis 2: Eligible employees, who objectively have scarce time resources due to biographical and structural time rivalries, are more likely to participate in ALE because of the implementation of the *Bildungszeitgesetz* and the legal option to use paid working time to participate in ALE then employees who are untreated by the law.

## Materials and Methods

Our study on effects of the implementation of the Bildungszeitgesetz belongs to the area of policy implementation research (Weimer and Vining, 2005; Damschroder et al., 2013; Heid, 2015). This requires a special focus on the causal relationship between the intervention and its consequences. Current academia regarding the requirements on data and methods to estimate such causal effects (Morgan and Winship, 2007; Schneider et al., 2007; Blossfeld et al., 2009; Murnane and Willett, 2011; Schlotter et al., 2011) agrees that “conditioning techniques on observable variables in cross-sectional settings is a rather weak approach to estimating causal effects” (Strietholt et al., 2013, p. 569). For this reason, we use longitudinal data and methods to estimate causal inference. According to the data and methods requirements formulated by current policy implementation research, our research can be classified as use-inspired basic research (Stokes, 1997; Towne and Shavelson, 2002; Fischer et al., 2005). Not only do we apply scientific methods to investigate effects of an educational policy intervention in order to determine whether the intended objectives of the intervention are actually achieved (Leutner, 2013), but we aim at drawing causal inferences based on theoretical assumptions. By estimating theory-based causal effects on specific subpopulations, we can additionally generate knowledge about the conditions of the implementation and identify subpopulations that benefit most from the implementation of the *Bildungszeitgesetz*. Thereby, we intend to generate knowledge examining effects of the implementation by testing broad theories (Lauen and Tyson, 2009), but also to generate knowledge on the effectiveness of the implementation of educational policy interventions (Campbell and Levin, 2009; Wiseman, 2010; Slavin, 2016).

All statistical analyses were performed with stata. The corresponding do-file can be found on OSF^1^.

### Data

The current database regarding laws on educational leave in Germany is limited and the data available for various aspects of this educational policy intervention (e.g., supply, exact number of participation, subjects and costs) are insufficient. Accordingly, the current situation in Germany cannot be presented systematically and overall statements regarding participation rates in educational leave remain uncertain (Grotlüschen and Haberzeth, 2018). As a result, in the perspective of policy implementation research, no treatment effects toward causal inference are estimated.

**FIGURE 1:**
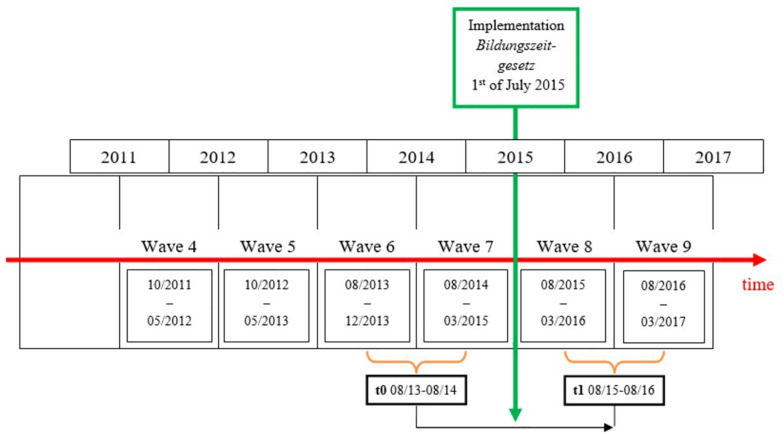
Definition of two periods as the timeframe of our analysis.

To tackle this weak and insufficient database, we decided on empirically using longitudinal data from the National Educational Panel Study (NEPS)^2^ in Germany (Blossfeld et al., 2011). Large-scale datasets such as the NEPS as nationally representative survey data are considered the most valid, rigorous and reliable source of evidence in the context of educational policy research (Lauen and Tyson, 2009). Given the fact that effects of educational policy interventions develop over the time period after its implementation (Hasselhorn et al., 2014), longitudinal research designs that allow causal analyses of such effects on participation in ALE represent a suitable methodological approach. Thus, the demand for longitudinal designs and evidence-based research (Desjardins and Schuller, 2006; Strietholt et al., 2013; Roßbach and Maurice, 2018) can be realized. NEPS data are especially suited for our purpose for two reasons. First, the NEPS data structure allows us to construct a counterfactual design of two experimental groups, and thereby differentiate between treated and untreated individuals by the *Bildungszeitgesetz*. Second, we can reconstruct individual participation in ALE of both groups over time. Using the NEPS data, we can compare trajectories regarding participation in ALE of both experimental groups. Moreover, we can estimate causal effects on individual trajectories caused by the implementation of the law. The NEPS is a multi-cohort sequence panel in which since 2007 approximately 60,000 individuals in ten waves are questioned about their educational behavior as well as their socio-economic and demographic background. The cohort sampling of the NEPS is based on six phases of the learning biography: Early childhood, kindergarten, fifth-graders, ninth-graders, first year students and adults. We use the sixth start cohort (Adults) focusing on lifelong learning and adult education. In this cohort, a total number of 17,140 individuals of the birth years 1944–1986 living in private households in Germany was interviewed (Stöckinger et al., 2018). With individual unit non-response, these are 91,864 observations. Using the NEPS data, we define two periods as the relevant timeframe for our analysis as shown in Figure 1: One timeframe before the implementation took place (t0) and one timeframe in which eligible employees are treated (t1). We define the t1 period directly after the implementation of the law in July 2015 because we assume that the salience of the law to be at its highest level at this point. To support the assumption regarding the salience of the law, we used the Google internet information source Google Trends^3^ to obtain information regarding actual searches on keywords of interest in the timeframe of our study. The results of Google Trends provide “a time series index of queries users enter into Google in a given geographic area” where the “maximum query share in the time period specified is normalized to be 100” (Choi and Varian, 2012, p. 3). In this study, we entered the following keywords into the program, where at the same time specifying the origin of the queries (Germany as a whole country or Baden-Württemberg as a German federal state) in which the keywords were most popular within the specified period of the last 5 years: *Bildungszeitgesetz Baden-Württemberg*; *Bildungszeitgesetz*; *Bildungszeit Baden-Württemberg*; *Bildungsurlaub Baden-Württemberg*. From Google Trends, we obtained the information that the highest interest in the keywords as the maximum query share of 100 is identified for the periods shortly before (e.g., *Bildungsurlaub Baden-Württemberg*: 08.-14. March 2015), during (e.g., *Bildungszeitgesetz*: 28.06.-04.07.2015) or after the implementation of the law on educational leave (e.g., *Bildungszeitgesetz Baden-Württemberg*: 22.-28.11.2015). These results generally support our assumption regarding the salience and the definition of the two periods as the relevant timeframe of our study.

### Description of the Sample

Using the NEPS data, we can precisely identify employees who are eligible according to the *Bildungszeitgesetz* in Baden-Württemberg. These are employees whose main working place is located in Baden-Württemberg and whose employment status exists for at least 12 months in the same company (§4 BzG BW). Our control group consists of all individuals, in Baden-Württemberg as well as whole Germany, who are not eligible. Since a similar law was introduced in the German federal state of Thuringia in 2016, we had to exclude employees working in Thuringia from our final sample. After this restriction, our final sample included 15,224 observations in the treatment and control group, as shown in Table 1. This results in a sample of *N* = 7,612 individuals whose course participation is in accordance with the specified content eligible to the law in both periods t0 and t1; with *n* = 709 treated by the law and *n* = 6,903 not treated by the law.

**TABLE 1:** Descriptive statistics and labeling of model-specific pretreatment variables.

	**Control (*n* = 6,903)**			**Treated (*n* = 709)**		
	***M***	***SD***	***Min/Max***	***M***	***SD***	***Min/Max***
**Continuous variables**						
Year of birth	1962.9	10.738	1944/1986	1964.5	9.431	1944/1986
Years of the educational experience (CASMIN)	14.203	2.355	9/18	14.631	2.361	9/18
Number of children in the household	0.754	0.976	0/6	0.987	1.106	0/9

	***%***	***M***	***SD***	***%***	***M***	***SD***

**Categorical variables**						
Gender		1.516	0.499		1.451	0.497
Male (1)	48.36			54.87		
Female (2)	51.64			45.13		
Status of Migration		0.840	0.365		0.777	0.416
No Migration (0)	84.09			77.72		
Migration (1)	15.91			22.28		
Net household income		6.518	2.013		7.262	1.728
<500 Euro (1)	0.65			0.56		
500–1,000 (2)	3.26			0.42		
1,000–1,500 (3)	6.33			2.68		
1,500–2,000 Euro (4)	7.81			4.51		
2,000–2,500 Euro (5)	11.62			9.31		
2,500–3,000 Euro (6)	11.13			8.04		
3,000–4,000 Euro (7)	23.67			23.41		
4,000–5,000 Euro (8)	15.86			19.46		
>5,000 Euro (9)	19.67			31.59		
Marital status						
Married/in registered partnership (1)	71.55			74.33		
Divorced (3)	10.00			8.89		
Widowed (4)	3.46			3.24		
Single (5)	14.99			13.54		
Children		0.449	0.497		0.551	0.497
No Children (0)	55.01			44.85		
Children (1)	44.99			55.15		
Single parent		0.0671	0.250		0.0789	0.269
In a partnership, with or without child/children (0)	93.29			92.10		
without partnership, with child/children (1)	6.71			7.90		

*All variables are measured before the treatment. *Source*: NEPS (Blossfeld et al., 2011), own calculations.*

In order to estimate causal effects of the treatment implementation on individual participation in ALE, descriptive analysis offer a good starting point for participation rates and changes in attended courses over time. The depending variable is the difference between the numbers of attended courses in ALE in the observed period t0 and t1, insofar as the content of the course is eligible according to the *Bildungszeitgesetz*. Table 2 provides descriptive analysis of the number of attended courses for both experimental groups.

**TABLE 2:** Descriptive statistics of the outcome variable.

		**Control**			**Treated**		
		***M***	***SD***	***Min/Max***	***M***	***SD***	***Min/Max***
**Outcome variable**							
Courses	Overall	0.584	1.111	0/13	0.806	1.340	0/9
	Between		0.914	0/9.5		1.086	0/6
	Within		0.631	−4.415/5.584		0.785	−3.192/4.806
			*N* = 13,806			*N* = 1,418	
			*n* = 6,903			*n* = 709	
			*T* = 2			*T* = 2	

*Source: NEPS (Blossfeld et al., 2011), own calculations.*

The available case numbers are thus also higher than in the corresponding evaluation study (f-bb, 2019), in which *n* = 535 eligible employees were surveyed.

### Data Analysis

#### Difference-in-Differences Estimation Strategy

Since our treatment cannot be randomly assigned to different experimental groups, we performed our study in a quasi-experimental research design in a natural setting. The non-randomization to the treatment can lead to selection bias when using standard routines such as least squares regression to estimate causal effects (Hogan and Lancaster, 2004). Thus, causal effects of the treatment may be confounded by differences in background characteristics (Foster, 2010). In order to estimate causal effects of the implementation of the *Bildungszeitgesetz*, we employ a difference-in-differences (DID) estimation strategy, treating the implementation of the *Bildungszeitgesetz* as a plausible source of exogenous variation (Tandberg and Hillman, 2014). This approach requires the definition of two periods t0 and t1 and an identification of one group that received the treatment and one group whose individuals remain untreated by the intervention (Heckman et al., 1997; Heckman et al., 1998; Athey and Imbens, 2006). The DID estimator “measures the impact of the program intervention by the difference in the before-after change in outcomes between participants and non-participants” (Todd, 2008, p. 3857). We can interpret this difference as the causal effect of the treatment on the outcome (Angrist et al., 1996).

A common criticism regarding low participation rates in educational leave is an unawareness of the legal entitlement of eligible employees (Grotlüschen and Haberzeth, 2018). We considered this question of how well informed the eligible employees are about educational programs and courses in a separate analysis and re-estimated the following models with a specification of the information status. We identified employees with a high level of information and therefore, with a higher probability of being aware of their eligibility status regarding the *Bildungszeitgesetz* with regard to their self-assessment in the NEPS to the question (t31461a): “How well do you know courses, offered by educational institutions with job-related content?” Beyond this self-assessment, there are nether further information in the dataset indicating the circumstances of the actual level of information nor information on by whom the individual has been informed about courses. With regard to our analysis, we define those employees as being informed, who have answered this question with very good, rather good or partial/partial. Because of this restriction, the number of observations changes. The treatment group now consists of *n* = 534 individuals for t0 and t1 and the control group consists of *n* = 3999 individuals.

#### Propensity Score Matching

In order to control for both observable and unobserved baseline differences, we apply a difference-in-differences propensity score matching (DID-PSM).

Following the counterfactual logic and the common trend assumption, inter- and intrapersonal comparisons within the treatment and control group allow us to eliminate unobserved baseline differences and allows for selection of unobservables (Todd, 2008; Gebel and Voßemer, 2014). In order to make the common trend assumption more plausible, we create the control group based on the probability of being eligible to the *Bildungszeitgesetz*. Rosenbaum and Rubin (1983) define this probability of assignment to the treatment as the propensity score. The propensity score is the conditional probability of the assignment to the treatment due to individual pretreatment characteristics. It is denoted as *P*(*X*) = Pr (*D* = 1|*X*), where *D* = {0,  1} is the indicator of assignment to the treatment and *X* is the multidimensional vector of pretreatment measured individual characteristics. The first step of the PSM is the estimation of the individual propensity score that predicts the probability of being assigned to the treatment. We used a logit regression to estimate the assignment to the treatment as a function of observable pretreatment characteristics (covariates) shown in Table 3 in order to avoid endogeneity problems:

**TABLE 3:** Covariates of the propensity score matching model.

**Treatment**	***Coef.***	***SE***	***Z***	***P*>*|z|***
**Covariates**				
Year of birth	0.006	0.003	2.83	0.005
Gender	–0.112	0.041	–2.71	0.007
Years of the educational experience (CASMIN)	0.006	0.009	0.66	0.511
Status of Migration	0.235	0.051	4.56	0.000
Net household income	0.109	0.013	8.41	0.000
Marital Status (Ref.: single) married/in registered partnership)	–0.0395	0.067	–0.59	0.556
Marital Status (Ref.: single) divorced	0.109	0.091	1.20	0.232
Marital Status (Ref.: single) widowed	0.223	0.132	1.68	0.093
_cons	–13.837	4.257	–3.25	0.001

*All covariates are measured before the treatment. “Ref.”, reference group. *Source*: NEPS (Blossfeld et al., 2011), own calculations.*

Step two is the formation of “statistical twins” (Gebel and Voßemer, 2014, p. 131) from both treatment and control group. We used the kernel-matching algorithm as a weighting method based on the propensity score. By using the kernel-matching algorithm, all treated individuals are matched with a weighted average of all individuals in the control group (Frisco et al., 2007). Applying PSM allows us to estimate the average treatment effect on the treated (ATT). We define the ATT as the effect of the implementation of the law on educational leave on the number of attended courses for those individuals who actually received the treatment:

$$A\,T\,T^{D\,I\,D-P\,S\,M}=\frac{1}{N_{D_{1}}}\,\sum_{i\in D_{1}\,\cap S}\left[\left(Y_{i,t+1}^{1}-Y_{i,t}^{0}\right)\,-\sum_{j\in D_{0}\,\cap S}w_{i\,j}\,\left(Y_{j,t+1}^{0}-j_{i,t}^{0}\right)\right]$$

where *D*_*1*_ represents the treatment group and *D*_*0*_ the control group, *w*_*ij*_ the kernel-matching weights and *S* the area of common covariate support (Gebel and Voßemer, 2014). Since the propensity score only uses family status and the number of children, we can only test hypothesis 1 with this approach.

#### Instrumental Variable

The DID-PSM approach intends to reduce selection bias in the assignment to the treatment based on observable differences and unobserved baseline differences. The key assumption is, that because the estimation of the propensity score is based on observed baseline covariates, individuals with a similar propensity score will have similar baseline covariates and thus are comparable (Morgan, 2018). Nevertheless, strategies on estimating causal effects of a treatment on an outcome in a quasi-experimental design may be biased in case of selection-on-unobservables (Heckman, 1977) and heterogeneous treatment effects (Heckman et al., 2006). The problem is, that when unobserved factors significantly affect the non-random assignment to the treatment, estimation strategies relying on observables can no longer estimate causal effects consistent and the estimator may be biased (Cerulli, 2015). This issue may be caused by the existence of endogenous explanatory variables correlated with the unexplained part of the dependent variable (Wooldridge, 2002; Bollmann et al., 2019). Besides the hypothesis of selection-on-unobservables, there is the possibility of heterogeneous treatments bias (Heckman et al., 2006). This relates to the possibility that individuals may respond differently to the treatment depending on their baseline characteristics. In our study, we assume heterogeneous response to the treatment depending on observed baseline differences but further assume unobserved effect heterogeneity. One standard method in social science and empirical economics to reduce or to eliminate this bias in case of selection-on-unobservables and heterogeneous treatments effects is to apply an instrument variable (IV) estimator (Angrist et al., 1996; Heckman, 1997; Wooldridge, 2010). Currently, instrument variables are commonly used in different disciplines like economics (Angrist and Krueger, 1991; Card, 2001; Pischke and Wachter, 2008), political science (Kern and Hainmueller, 2009; Hansford and Gomez, 2010) also but rarely in educational science (O’Connell et al., 2017) and in psychology (Bollmann and Krings, 2016; Bollmann et al., 2019).

The most important condition for an instrument is that it correlates with the treatment, but not with the error term. Thus, random events or characteristics correlated with the treatment are the safest exogenous instruments. Following this condition, we use the birthplace of Baden-Württemberg as such an instrument. We assume that the place of birth is at random and not correlated with the error term, but with the treatment. We code the instrument *Z* as a binary variable.*Z*is coded to take two values *Z*={0, 1} indicating the birthplace in Baden-Württemberg (1) or elsewhere in Germany (0). The treatment variable *Bildungszeitgesetz* is also binary coded*D*={0,1}.

In case of a binary treatment, we used a probit two stage least square model (probit 2SLS) to estimate the treatment effect on the outcome*Y*. The probit 2SLS allows estimating consistent average treatment effects overall (ATE), on the treated (ATT) and on the non-treated (ATNT) under the hypothesis of selection-on-unobservables and heterogeneous treatment effects. Operationally, the probit 2SLS follows three steps (Cerulli, 2014):

$$y_{i\,t\,1}-y_{i\,t\,0}=\beta_{0}+\beta_{1}\,D_{i}+\sum \beta \,x_{i}+\varepsilon_{i}$$

$$D_{i}=a_{0}+a_{1}\,{\hat{D}}_{i}+a_{2}\,Z_{i}+u_{i}$$

First, we estimate a probit regression of the treatment *D* on *X* and *Z*, thus estimating the predicted probability of assignment to the treatment $\hat{D}$. We predicted the values of $\hat{D}$ as a function of observable pretreatment characteristics (covariates) and the instrument. Second, we used these predicted probabilities of assignment to the treatment D as an instrument by applying a two stage least square (2SLS) (Angrist and Imbens, 1995; Heckman, 1997; Angrist and Pischke, 2009). The outcome is the first difference of the number of attended courses. Table 4 covers the covariates and the instrumental variable of the probit model:

**TABLE 4:** Covariates and the instrumental variable in the probit model.

**Treatment**	***Coef.***	***SE***	***Z***	***P* > *|z|***
IV	2.081	0.056	37.39	0.000
Year of birth	0.009	0.003	3.35	0.001
Gender	–0.2001	0.051	–3.94	0.000
Years of the educational experience (CASMIN)	0.010	0.011	0.90	0.368
Status of Migration	0.361	0.060	6.02	0.000
Net household income	0.088	0.016	5.38	0.000
Marital Status (Ref.: single) married/in registered partnership	0.031	0.095	0.33	0.745
Marital Status (Ref.: single) divorced	0.203	0.112	1.81	0.071
Marital Status (Ref.: single) widowed	0.173	0.166	1.05	0.295
Children	0.014	0.064	0.22	0.827
Single parent	0.141	0.124	1.1	0.254
_cons	–20.706	5.479	–3.78	0.000

*All covariates are measured before the treatment. “Ref.”, reference group. *Source*: NEPS (Blossfeld et al., 2011), own calculations.*

With this approach we test hypotheses 1 and 2. To test hypothesis 2 we used the conditional effects of the treatment and the single parent characteristic (without partnership, with child).

## Empirical Results

### Propensity Score Model

We tested the average treatment effect on the treated in a comparison consisting of eligible employees to the *Bildungszeitgesetz* and those adults that are untreated in Baden-Württemberg and Germany as a whole. In order to assess the effect of the implementation of the *Bildungszeitgesetz* on individual participation in ALE, descriptive analyses offer a good starting point. Table 5 displays the changes in the average number of attended courses between the respective treatment and control group and the difference of the differences, which measures the impact of the implementation.

**TABLE 5:** Difference-in-differences estimation results.

**Outcome variable**	***Ø number of attended courses***	***SE***	***Z***	***P*>*|z|***
Before				
Control	0.726			
Treated	0.908			
Difference (T-C)	0.182	0.029	6.38	0.000***
After				
Control	0.549			
Treated	0.705			
Difference (T-C)	0.156	0.029	5.48	0.000***
Difference-in-Differences	–0.026	0.040	0.63	0.526

*NEPS (Blossfeld et al., 2011), own calculations. ^∗∗∗^*p* < 0.01.*

The results generally reveal changes in the average number of attended courses in ALE both in the treatment group and in the control group between t0 and t1. Furthermore, the results reveal a baseline difference in the average number of attended courses in ALE of 0.182. This shows that individuals who are eligible to the *Bildungszeitgesetz* already attend more courses in ALE than those who are not eligible to the law. This baseline difference remains significant at 0.156 even after the implementation of the *Bildungszeitgesetz* in t1. Overall, we can observe a negative trend regarding inter- and intrapersonal comparisons of the average number of attended courses for both experimental groups. The intrapersonal comparison reveals a difference of −0.203 for the treatment group and for the control group −0.177.

To estimate the effect of the treatment on the outcome, we can interpret the difference in the before-after change in outcomes between participants and non-participants as causal (Angrist et al., 1996). The result of the difference of the differences between t0 and t1 reveals no significant average treatment effect on the treated (ATT). Our results even point to a slightly negative difference of −0.026 between the differences.

With regard to the informed eligible employees, the results confirm the empirical finding of a baseline difference in the average number of attended courses in ALE. The results show that on average, informed eligible employees participate in 1.028 educational activities at t0 and of 0.845 at t1. In comparison to this, non-eligible informed employees participate less in educational activities with an average of 0.886 course at t0 and 0.694 at t1. Compared to the first model, both values are higher at both times. Thus, the results show that well-informed employees generally have a higher level of participation in ALE.

In comparison to the model without the specification of the information status on offered programs in ALE with job-related content, the difference of the differences in the average number of attended courses is 0.008, whereas in the previous model, the difference of the differences is -0.026. Conclusively, when taking the status of being informed about educational programs and courses with job-related content into account, the result of the difference of the differences between t0 and t1 also reveals no significant ATT of the implementation of the *Bildungszeitgesetz* on the difference of the number of courses attended.

### Instrumental Variable Model

In a second approach, we applied an IV in a probit 2SLS model to estimate the causal effect of the implementation of the *Bildungszeitgesetz*. We report the main outcome of the 2SLS below, where the results from both the probit first-step regression and the IV regression of the second step are revealed. Results on the probit in Table 6 show that $\hat{D}$ is highly correlated with the treatment, thus we can use it as an instrument for this variable.

**TABLE 6:** First stage regression.

	***Coef.***	***SE***	***t***	***t*>*|z|***
**Treatment**				
Year of birth	0.0008	0.0003	0.57	0.567
Gender	–0.0023	0.0059	–0.39	0.693
Years of the educational experience (CASMIN)	–0.0005	0.0012	–0.42	0.677
Status of Migration	–0.0004	0.0086	–0.06	0.956
Net household income	0.0006	0.0018	0.35	0.730
Marital Status (Ref.: single) married/in registered partnership	0.0031	0.01001	0.31	0.754
Marital Status (Ref.: single) divorced	0.0022	0.0120	0.19	0.852
Marital Status (Ref.: single) widowed	0.0021	0.0172	0.12	0.902
Children	0.0008	0.0070	0.11	0.915
Single parent	0.0047	0.0137	0.34	0.732
$\hat{D}$	0.9184	0.1042	8.81	0.000
IV	0.0464	0.0591	0.79	0.432
_cons	–0.34868	0.6217	–0.56	0.575

*“Ref.”, reference group. *Source*: NEPS (Blossfeld et al., 2011), own calculations.*

In the second stage shown in Table 7, we used the predicted values in a standard OLS regression of *Y* on $\hat{D}$. That is, the difference of the number of attended courses *Y* was regressed on the predicted values of assignment to the treatment *Bildungszeitgesetz*.

**TABLE 7:** Instrumental variables (2SLS) regression.

**Y (outcome)**	***Coef.***	***SE***	***t***	***t*>*|z|***
Treatment	0.066	0.0764	0.86	0.387
_ws_Treatment	0	(omitted)		
_ws_Year of birth	–0.0123	0.0123	–1.01	0.313
_ws_Gender	0.0019	0.1762	0.01	0.991
_ws_Years of the educational experience (CASMIN)	0.0039	0.0412	0.09	0.925
_ws_Status of Migration	–0.5663	0.2414	–2.35	0.019
_ws_Net household income	0.0109	0.0584	0.19	0.852
_ws_married/in registered partnership	–0.4421	0.3686	–1.20	0.230
_ws_divorced	0.0770	0.4498	0.17	0.864
_ws_widowed	0.3739	0.5929	0.63	0.528
_ws_Children	0.2021	0.2209	0.91	0.360
_ws_ Single parent	–0.2987	0.4549	–0.66	0.511
Year of birth	–0.0001	0.0019	–0.03	0.973
Gender	–0.0261	0.0339	–0.77	0.441
Years of the educational experience (CASMIN)	–0.0126	0.0079	–1.60	0.109
Status of Migration	0.0420	0.0492	0.85	0.393
Net household income	–0.0303	0.0099	–3.04	0.002
Marital Status (Ref.: single) married/in registered partnership	0.1643	0.0629	2.61	0.009
Marital Status (Ref.: single) divorced	0.1470	0.0739	1.99	0.047
Marital Status (Ref.: single) widowed	0.0404	0.1060	0.38	0.703
Children	0.0188	0.0447	0.42	0.674
Single parent	0.0727	0.0850	0.86	0.392
_cons	0.22878	3.785	0.06	0.952

*“Ref.”, reference group. *Source*: NEPS (Blossfeld et al., 2011), own calculations.*

**FIGURE 2:**
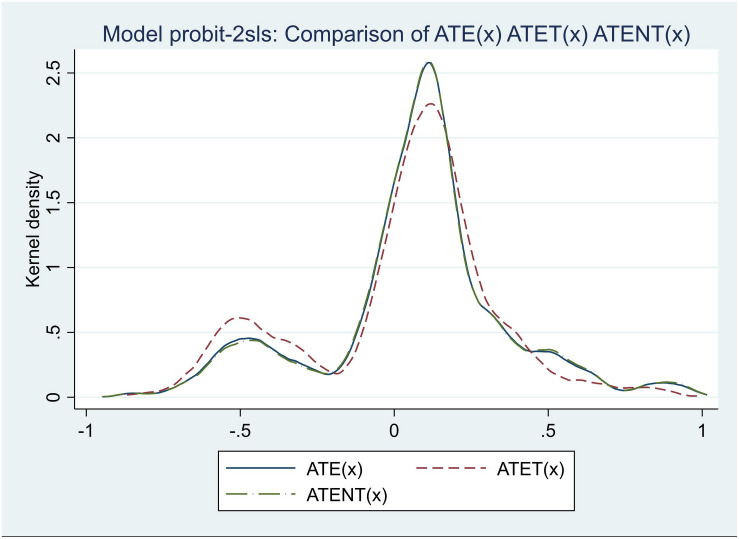
Monte-Carlo-simulation comparing the cross-unit distribution of ATE(x), ATET(x), and ATENT(x).

Step two shows that the average treatment effect (ATE) of the treatment *D* on the outcome *Y* is positive, but not significant (*p* = 0.066). These results lead to a rejection of hypothesis 1. Having assumed heterogeneous responses to the implementation of the *Bildungszeitgesetz* on an individual level, we can further calculate the average treatment effect on the treated (ATET) and the average treatment effect on the non-treated (ATENT) and therefore analyze the cross-unit distribution of ATE(x), ATET(x), and ATENT(x) in a Monte-Carlo-simulation (Cerulli, 2014), as shown in Figure 2.

We see that ATET(x), ATET(x), and ATENT(x) present a substantially uniform distribution. In particular, all three distributions show the highest modal value around 0.021. We also find a high negative value around −0.5. In order to analyze the treatment effect, it is particularly interesting to identify the individuals who negatively benefited from the implementation of the *Bildungszeitgesetz*. In order to differentiate the distribution of the ATE(x), we can further calculate the conditional effect of the treatment for specific subpopulations with a specific characteristic. The distribution with a negative treatment effect represents the 25% percentile. At this point, we can compare values of the selected covariates for those individuals for whom the ATE is positive (*n* = 5,709) with those, for whom the ATE is negative (*n* = 1,903), as shown in Table 8.

**TABLE 8:** Comparison of average treatment effects.

**Variable**	***M***	
	**Positive ATE**	**Negative ATE**
Year of birth	1961.119	1968.842
Gender	1.492	1.565
Years of the educational experience (CASMIN)	14.221	14.308
Status of Migration	0.0105	0.628
Net household income	6.575	6.628
Single parent	0.079	0.0342

*Source: NEPS (Blossfeld et al., 2011), own calculations.*

The results of this comparison reveal detrimental effects special subpopulations caused by the implementation of the *Bildungszeitgesetz*. These individuals are on average 7 years younger, are more women and are significantly more migrants. We can see that only 1.05% of the individuals with a positive ATE are migrants, whereas 62.8% of the individuals who benefited negatively are migrants. Single parents, on the other hand, have no significant advantage. Their proportion among those with positive ATE is higher, but the conditional effect in the model is not significant.

Using an IV is based on several conditions (Hall et al., 1996; Stock and Watson, 2003; Angrist and Pischke, 2009). First, the instrument relevance condition must be met, meaning that the instrument *Z* is correlated with the treatment variable*Dcov*(*Z*,*D*)≠0. The second condition, the instrument exogeneity condition, implies that at the same time *Z* is not correlated with the error term of the model*cov*(*Z*,*u*) = 0. We can verify the instrument relevance condition by testing the strength of the correlation between *Z* and *D*. In this case, we use the standard first stage F-statistic to determine whether the instrument has sufficient explanatory power (Staiger and Stock, 1997). The *F*-value of our instrument at 2004.02 exceeds the threshold of ten (Stock and Yogo, 2005). Based on this, we can assume that the instrument is strong and satisfies the relevance condition. With regard to the instrument exogeneity condition, we can only theoretically explain that*Z* is not correlated with the error term*u*, since we cannot test the condition statistically. We strongly assume that there is no correlation of the birthplace with the dependent variable. Because the theoretical identified instrument ‘birthplace’ satisfies both formulated conditions, we state that *Z* is a valid exogenous instrument for *D*.

The last step in our analysis is to check if there is an endogeneity problem by performing a Wu-Hausmann test. For our model, the test was not significant (*p* = 0.293) with a value of 1.107. Therefore, we cannot reject the *H*_0_. This indicates that our estimation was not biased by endogeneity.

With regard to the informed eligible employees, the results show that the average treatment effect (ATE) of the treatment *D* on the outcome *Y* has a positive, but still non-significant value (*p* = 0.079). In comparison to the model without the specification of the information status on courses in ALE with job-related content, the ATE is slightly higher, but still not significant. In terms of an evaluation and the associated questions of the impact and effectiveness of education policy interventions, a *p*-value above 0.05 is not sufficiently restrictive.

## General Discussion

The aim of our study was to estimate the causal effect of the implementation of the *Bildungszeitgesetz* in the German federal state of Baden-Württemberg in 2015 on individual participation in ALE of eligible employees. Our analyses reveal that we cannot confirm the theoretical assumption that the empowerment of employees to claim educational leave from their employers leads to a higher number of attended courses in ALE (Hypothesis 1). In consequence, our research design applied to our dataset cannot support the assumption that the availability of time as a resource is as relevant in the decision to participate in an educational activity as suggested by the MFS (Kroneberg, 2005, 2011) and the SEU-theory (Esser, 1993, 1999).

Proceeding the rejection of hypothesis 1, questions regarding possible reasons for the ineffectiveness of the implementation of the *Bildungszeitgesetz* on individual participation in ALE arise. To answer these questions, we focus on the mechanisms and conditions by which the educational policy intervention is implemented (Damschroder et al., 2013; Heid, 2015). We identify three possible reasons for the ineffectiveness of the law in our study design: Deadweight effects, zero-sum effects and unconsidered costs.

The results of the DID-PSM analysis revealed significant baseline differences between eligible and non-eligible individuals in the average number of attended courses in ALE. This difference illustrates that the *Bildungszeitgesetz* addresses those individuals that already have higher participation rates in ALE compared to the non-eligible individuals. As a result, the probability of windfall effects increases: Already intended participation in ALE is now realized through an educational leave. Concluding, the implementation of the *Bildungszeitgesetz* causes no additional participation in ALE, as there is no significant difference of the number of attended courses among the treatment group and control group. This leads to the conclusion, that a specific educational activity would probably also have taken place without the *Bildungszeitgesetz* because of the higher affinity toward educational activities and higher motivation to participate in ALE.

The second reason for the possible ineffectiveness of the law focuses on the imposing of obligations on the employer regarding the exemption and continued payment of salary. The conditional effects of the probit 2SLS revealed detrimental effects for some subgroups. As a result of the implementation, the average number of attended courses for these individuals has even decreased. These findings highlight that because of the implementation of the law, younger adults, women and individuals with a migration background in particular are confronted with additional disadvantages, whereas only the results of migrants are statistically significant. This result is insofar of particular importance, as according to Siebert (2015), the effectiveness of a law on educational leave has to be evaluated primarily based on the extent to which educationally disadvantaged individuals are reached in terms of equal opportunities. This suggests that the advantages of some are bought by the disadvantages of others. With regard to the employers, it is conceivable that for example employers might reduce their own financial support of participation in ALE in terms of an external selection when it is legally imposed to grant educational leave. This applies particularly to those individuals who may apply for educational leave to a lesser extent: Migrants, for example.

Moreover, the results of the probit 2SLS are interesting for another reason. Current results of the AES in 2016 highlight that women and adults with a migration background experience an increasing number of discontinuous employment biographies and as a result, participate to a higher degree in job-related and non-job-related non-formal education and training to obtain knowledge or to learn new skills needed for a current or future job (Kuper et al., 2017). These are the segments of ALE, in which laws on educational leave intend to foster and support participation. The empirical results of our study reveal, however, that the implementation of the *Bildungszeitgesetz* has not been able to reach these groups of adults and increase their participation in ALE. In fact, the implementation even has a detrimental effect on participation in ALE for these subpopulations.

Finally, the third reason refers to possible additional hidden costs, which may exceed the initial benefit of the *Bildungszeitgesetz* and the availability of time resources due to the implementation. Particularly in modern knowledge-based services, independent understanding and representation of company interests (consummate cooperation) is a decisive factor for productivity (Williamson, 1985). In order to participate in an educational activity, legitimated by the *Bildungszeitgesetz*, employees have to assert their interests against those of the employer. The *Bildungszeitgesetz* empowers employees to do this and to claim educational leave, but at the same time, they must fear to suffer informal disadvantages by their employer. The idea that employees might avoid this possible confrontation is also reflected in the results that younger people, women and migrants in particular benefit less from the *Bildungszeitgesetz*. Because of potential disadvantages such as a weaker standing in the company or worse labor market opportunities, it is conceivable to assume that employees avoid acting against the interests of their employer and therefore avoid to claim educational leave.

### Limitations

Although the present research contributes to the literature as the first study estimating causal effects of the implementation of a law on educational leave in Germany, several limitations need to be taken into account when assessing the empirical findings on effects of the implementation of the *Bildungszeitgesetz* on individual participation in ALE.

The first limitation relates to the definition of the two periods as the timeframe for our analysis. We observed individual participation in ALE for both experimental groups 1 year before (t0) and 1 year after (t1) the implementation of the *Bildungszeitgesetz*. Both, the assumption that the salience is at its highest at the time of the implementation of the law on educational leave and the associated information obtained from Google Trends regarding queries for defined keywords in the context of the *Bildungszeitgesetz* support the definition of our timeframe. However, we did not investigate effects of the implementation on participation in ALE beyond that specific timeframe. Since effects of interventions on the level of teaching and learning processes (micro-level) as well as on the level of organization institutions like educational institutions but also companies (meso-level) develop over time after the implementation (Hasselhorn et al., 2014). Thus, our defined timeframe could be too short to observe changes. Following our results, the models would have to be re-estimated over a longer period, while at the same time focusing on further mechanisms of the implementation on the micro- and meso-level. In addition, surveys of companies and educational institutions could be used to investigate whether and to what extent the implementation of the *Bildungszeitgesetz* changes the ‘learning culture’ of companies or the structure of the offered educational programs. These questions could further be answered by investigating effects of the implementation on the process of program-planning as well as on the structure of offered programs. Furthermore, a subsequent question deals with the mechanisms that determine the successful implementation of laws on educational leave in the ‘learning culture’ of companies. Similar questions for the federal state of Bremen were already addressed in the study by Robak et al. (2015a). At this point, however, the questions for other federal states such as Baden-Württemberg remain unanswered. These further questions arise from the results of our study, but shift the attention to actors in the environment of eligible employees and their working-conditions at the meso-level, whereas our study focused on the direct causal effect of the implementation of the *Bildungszeitgesetz* on individual participation in ALE. These desiderata might pave the way for further research that complements our research by using broader methods, e.g., a mixed-methods design.

Secondly, we did not estimate effects of the implementation of the *Bildungszeitgesetz* on time spent in ALE as an outcome, as we operationalized participation in ALE as the number of attended courses. Further research should therefore focus on other measurements of participation in ALE, such as hours spent in educational activities. Nevertheless, when focusing on hours spent in educational activities, major problems arise regarding measurement errors and item non-response. Moreover, with regard to the measurement of hours spent in educational activities, it is conceivable to assume that the exact number of hours is not always correctly remembered. Furthermore, another problem concerns the estimation of the treatment effect of the implementation of the *Bildungszeitgesetz* itself, when operationalizing participation in ALE in hours. Although it is possible to assume effects of the treatment on the hours spent in ALE, the additional educational aspiration caused by the *Bildungszeitgesetz* is also expressed as the participation in another course. This is because the educational institutions already define the volume of hours in organized courses in their programs prior to the beginning of the course. In consequence, the operationalization of participation in ALE as the number of realized attended courses in our dataset is comparatively to the measured volume of hours to a higher level consistent, reliable and valid. The mentioned problems could bias the estimation of the true treatment effect because of insufficient and incomplete data.

The third limitation regards the interpretation of the estimated average treatment effect on the treated (ATT) by applying a DID-estimation strategy combined with an IV. We can only interpret the treatment effect for those eligible employees, for which the relationship between the estimated IV and the treatment applies. The causal effect of *D* on *Y* is therefore locally limited to a particular population with their birthplace located in Baden-Württemberg. We call this the Local Average Treatment Effect (LATE) (Angrist et al., 1996).

Furthermore, we re-estimated the DID-PSM and DID-IV models with a specification of the information status and the question of how well informed the eligible employees are about educational programs and courses. Further limitations result from the fact, that the NEPS data do not specify which level of information is exactly referred to in the question. Thus, even well informed employees may not have knowledge of the implementation of the *Bildungszeitgesetz* and the legal option to use working time as learning time.

Finally, there is a lack of any significant consideration of the educational leave in surveys such as the AES or the NEPS (Reichling, 2014). In future research, these surveys should also be used to investigate the participation in educational leave.

## Data Availability Statement

The datasets generated for this study are available on request to the corresponding author.

## Author Contributions

FR and AM contributed with the initial conceptualization of the study. FR conducted the deduction of theoretical assumptions as well as the empirical investigation. AM supervised the joint analysis of the data. All authors contributed to the revision of the final draft.

## Conflict of Interest

The authors declare that the research was conducted in the absence of any commercial or financial relationships that could be construed as a potential conflict of interest.

## Footnotes

1 https://osf.io/rbexw/?view_only=7916c2763e42471884dc8e07a70fae1f

2 This paper uses data from the National Educational Panel Study (NEPS): Starting Cohort Adults, 10.5157/NEPS:SC6:9.0.1. From 2008 to 2013, NEPS data was collected as part of the Framework Program for the Promotion of Empirical Educational Research funded by the German Federal Ministry of Education and Research (BMBF). As of 2014, NEPS is carried out by the Leibniz Institute for Educational Trajectories (LIfBi) at the University of Bamberg in cooperation with a nationwide network.

3 Google Trends. Available from: http://www.google.com/trends/.
