# Corrigendum: Educational Leave as a Time Resource for Participation in Adult Learning and Education (ALE)

**DOI:** 10.3389/fpsyg.2021.663146

**Published:** 2021-03-05

**Authors:** Fabian Rüter, Andreas Martin, Josef Schrader

**Affiliations:** German Institute for Adult Education (DIE), Bonn, Germany

**Keywords:** educational leave, time resources, rational choice, policy implementation research, evaluation

In the original article, there was an error.

In the Propensity Score Matching section of our manuscript, we described the rationale of applying a difference-in-differences estimation strategy with propensity score matching to estimate the causal effect of the implementation of the *Bildungszeitgesetz* on ALE participation. In our study, we defined the propensity score as the conditional probability of the assignment to the treatment (*Bildungszeitgesetz*) due to individual pretreatment characteristics. When revising our manuscript, we made a mistake on how to denote the propensity score. In line with the comments made by the editor and reviewers, we made some cuts to the text in the Propensity Score Matching section. Unfortunately, we did not completely delete parts of the explanations on how we denoted the propensity score. As a result, parts of the formula have been published incorrectly. Please find the corrected text for the Propensity Score Matching section below.

A correction has been made to *Data Analysis, Propensity Score Matching, Paragraphs 1 and 2*. The corrected text can be found below:

In order to control for both observable and unobserved baseline differences, we apply a difference-in-differences propensity score matching (DID-PSM).

Following the counterfactual logic and the common trend assumption, inter- and intrapersonal comparisons within the treatment and control group allow us to eliminate unobserved baseline differences and allows for selection of unobservables (Todd, 2008; Gebel and Voßemer, 2014). In order to make the common trend assumption more plausible, we create the control group based on the probability of being eligible to the *Bildungszeitgesetz*. Rosenbaum and Rubin (1983) define this probability of assignment to the treatment as the propensity score. The propensity score is the conditional probability of the assignment to the treatment due to individual pretreatment characteristics. It is denoted as *P* (*X*) = Pr (*D* = 1|*X*), where *D* = {0, 1} is the indicator of assignment to the treatment and *X* is the multidimensional vector of pretreatment measured individual characteristics. The first step of the PSM is the estimation of the individual propensity score that predicts the probability of being assigned to the treatment. We used a logit regression to estimate the assignment to the treatment as a function of observable pretreatment characteristics (covariates) shown in Table 3 in order to avoid endogeneity problems:

Additionally, in the original article, there was another error.

In the Empirical Results section, we present the estimation results for the Propensity Score Model and Instrumental Variable Model, respectively. In the Instrumental Variable Model section, we checked if there was an endogeneity problem by performing a Wu-Hausmann test. When revising our manuscript, we made some cuts to the text regarding the description of the Wu-Hausman test. Unfortunately, we did not completely delete parts in which we described the rationale of the Wu-Hausmann test as intended. As a result, parts of the description have been published incorrectly. Please find the corrected text below.

A correction has been made to *Empirical Results, Instrumental Variable Model, Paragraph 7*.

The last step in our analysis is to check if there is an endogeneity problem by performing a Wu–Hausmann test. For our model, the test was not significant (*p* = 0.293) with a value of 1.107. Therefore, we cannot reject the *H*_0_. This indicates that our estimation was not biased by endogeneity.

The authors apologize for these errors and state that they do not change the scientific conclusions of the article in any way. The original article has been updated.
